# The Reporting of Observational Clinical Functional Magnetic Resonance Imaging Studies: A Systematic Review

**DOI:** 10.1371/journal.pone.0094412

**Published:** 2014-04-22

**Authors:** Qing Guo, Melissa Parlar, Wanda Truong, Geoffrey Hall, Lehana Thabane, Margaret McKinnon, Ron Goeree, Eleanor Pullenayegum

**Affiliations:** 1 Department of Epidemiology and Biostatistics, McMaster University, Hamilton, Ontario, Canada; 2 Biostatistics Unit, St Joseph's Healthcare Hamilton, Hamilton, Ontario, Canada; 3 Department of Psychology, Neuroscience and Behaviour, McMaster University, Hamilton, Ontario, Canada; 4 Department of Psychology, University of Calgary, Calgary, Alberta, Canada; 5 Centre for Evaluation of Medicine, St Joseph's Healthcare Hamilton, Hamilton, Ontario, Canada; 6 Mood Disorders Program, St. Joseph's Healthcare Hamilton, Hamilton, Ontario, Canada; 7 Kunin-Lunenfeld Applied Research Unit, Baycrest, Toronto, Ontario, Canada; 8 PATH Research Institute, St. Joseph's Healthcare Hamilton, Hamilton, Ontario, Canada; 9 Department of Psychiatry and Behavioural Neurosciences, McMaster University, Hamilton, Ontario, Canada; Medical University of Vienna, Austria

## Abstract

**Introduction:**

Complete reporting assists readers in confirming the methodological rigor and validity of findings and allows replication. The reporting quality of observational functional magnetic resonance imaging (fMRI) studies involving clinical participants is unclear.

**Objectives:**

We sought to determine the quality of reporting in observational fMRI studies involving clinical participants.

**Methods:**

We searched OVID MEDLINE for fMRI studies in six leading journals between January 2010 and December 2011.Three independent reviewers abstracted data from articles using an 83-item checklist adapted from the guidelines proposed by Poldrack et al. (Neuroimage 2008; 40: 409–14). We calculated the percentage of articles reporting each item of the checklist and the percentage of reported items per article.

**Results:**

A random sample of 100 eligible articles was included in the study. Thirty-one items were reported by fewer than 50% of the articles and 13 items were reported by fewer than 20% of the articles. The median percentage of reported items per article was 51% (ranging from 30% to 78%). Although most articles reported statistical methods for within-subject modeling (92%) and for between-subject group modeling (97%), none of the articles reported observed effect sizes for any negative finding (0%). Few articles reported justifications for fixed-effect inferences used for group modeling (3%) and temporal autocorrelations used to account for within-subject variances and correlations (18%). Other under-reported areas included whether and how the task design was optimized for efficiency (22%) and distributions of inter-trial intervals (23%).

**Conclusions:**

This study indicates that substantial improvement in the reporting of observational clinical fMRI studies is required. Poldrack et al.'s guidelines provide a means of improving overall reporting quality. Nonetheless, these guidelines are lengthy and may be at odds with strict word limits for publication; creation of a shortened-version of Poldrack's checklist that contains the most relevant items may be useful in this regard.

## Introduction

In the past decade, the use of functional MRI (fMRI) studies in cognitive neuroscience has increased a great deal [1], [2]. Given that fMRI is increasingly applied to the study of clinical disorders (e.g., [3]–[8]), and considering the vulnerability of clinical participants, there is an ethical imperative for scientists to apply rigorous methodology and to provide adequate reporting. Rigorous methodology is required in order to uphold the promises typically made to participants during the consent process, namely that the study will help investigators to understand their conditions. Complete reporting with sufficient details permits readers to ensure the methodological rigor of a study [9], consider the validity of findings [10]–[14], and extend and replicate the findings [9]–[13], [15]–[17]. In particular, recent evidence indicates that overall, the fMRI literature lacks key details in their methods section, such as sample size calculations, whether temporal autocorrelations were modeled, descriptions of slice-timing and motion correction, slice order and coverage of functional brain images [18], and related parameter estimates (i.e., effect size and variance components) in the results section [19].

Standard guidelines have been developed to aid authors in reporting their research, such as the Consolidated Standards for Reporting Trials (CONSORT) [10] and the Strengthening the Reporting of Observational Studies in Epidemiology (STROBE) initiative [9]. Recently, Poldrack and his colleagues have proposed guidelines specifically for reporting fMRI studies [14]. Although many authors have suggested endorsing the guidelines proposed by Poldrack et al. in reporting fMRI studies to improve the quality, transparency and consistency of results [2], [18], [20], [21], few systematic reviews have been conducted to appraise the quality of reporting based on these guidelines. Although a study by Carp (2012) recently examined adherence to Poldrack et al.'s guidelines in randomly selected fMRI studies published since 2007, it included few studies involving clinical populations. Thus, the reporting quality in clinical fMRI studies remains unclear. Given the unique challenges (e.g., technical, interpretive, and methodological) that confront clinical fMRI studies, reporting details on design, subject characteristics, analyses and interpretation is suggested to enhance reproducibility of results in this subset of fMRI studies. Therefore, we expect that reporting in clinical fMRI studies is different from that of the overall fMRI literature.

Moreover, based on our experience and anecdotal evidence that the majority of fMRI studies are observational (i.e., the type of study is not designed to randomize participants to test efficacy and safety of any therapeutic intervention), these studies are less scrutinized than randomized clinical trials with experimental interventions; for example, randomized trials have to be registered with clinicaltrials.gov. Therefore, we aimed to systematically evaluate the quality of reporting in observational fMRI studies involving clinical human participants (i.e., individuals who either have a disease or are at risk of developing a disease) using a checklist adapted from the guidelines proposed by Poldrack et al. In this study, we set out to address the following two questions: (1) what percentage of articles reported each item of the fMRI-specific guideline, and (2) what percentage of items was reported per article?

## Methods

### Search Strategy and Eligible Journals

We searched OVID MEDLINE on January 2012 by using key word search terms (e.g., functional magnetic resonance imaging) combined with the acronym (i.e., fMRI) for articles published in 2010 and 2011, in the English language, and involving human participants. Compared with journals in general, top journals are cited more frequently (e.g., higher impact factors (IF)) and more scrutinized prior to publication (e.g., lower manuscript acceptance rates). Furthermore, studies have indicated that high IF and low manuscript acceptance rates of journals are associated with higher methodological rigor of articles published in the journals [22]–[26]. In this study, we further constrained our selection to six leading journals: In the Journal Citation Report 2010, we selected four journals with a high IF in the category “Neurosciences”, namely, Neuron (IF 14.9), Nature Neuroscience (IF 14.2), Brain (IF 9.2), Journal of Neuroscience (IF 7.3), one journal with the highest impact factor in the category “Neuroimaging” (NeuroImage, IF 5.94), and one journal which contributes a great number of articles in fMRI studies [18] and has a high impact factor (Proceedings of the National Academy of Sciences of the United States of America, IF 9.8). More details on the search strategy can be found on Table S1. Duplicate articles were removed.

### Eligibility Criteria for Studies and Study Selection

We included articles that were peer-reviewed, full reports of observational fMRI studies involving human clinical participants, and block or event-related or mixed design for the fMRI paradigm. We excluded articles that were published only in abstract form or any that were only editorials, letters, comments or reviews. Genetic, resting-state observational fMRI studies, fMRI studies other than observational studies (e.g., randomized clinical trials), and studies of connectivity were also excluded. As studies of connectivity aim to identify and quantify the correlations between brain regions [27], these studies have a different reporting focus vis-à-vis fMRI data analyses. For example, they report the Psycho-Physiological Interaction analyses to estimate effective connectivity or functional coupling rather than data preprocessing steps, which were demonstrated to have significant impacts on the quality of data and the reliability and interpretation of fMRI results [28] [29]. However, the reporting essentials for effective connectivity studies have not been reflected in the current available guidelines including the one proposed by Poldrack et al. As our study aimed to evaluate the quality of reporting based on Poldrack et al.'s guidelines, we therefore excluded this type of study to ensure consistency.

In this study, we decided to include a target sample size of 100 articles that had to meet the predefined inclusion and exclusion criteria. We therefore randomly selected and assessed the eligibility of articles among the unique citations, which were identified from the initial search strategy and after the duplicates were removed, until 100 articles were reached.

### Data Extraction

We created an electronic data extraction form containing 83 items adapted from the guidelines proposed by Poldrack et al. [14] to assess the reporting of study articles, which we piloted using a random selection of four studies reviewed by three independent reviewers (QG, MP, and WT). Through the pilot testing, we modified the abstraction form by deleting three items (Unwarping of B0 distortions; Describe any data quality control measures; any additional operations, e.g., masking out parts of the image) from Poldrack et al.'s original checklist. The reason for excluding these three items was that we found assessing them required too much subjectivity, meaning that biases among reviewers' judgments were very high. Excluding them meant we were better able to achieve a common perception and interpretation of definitions among items we did evaluate, and hence increased between-reviewer agreement. The observed percentage of agreement on judgments between any two reviewers was 0.78 or higher. Final abstraction forms were devised prior to use (see Table S2). The data were extracted from each article and any online supplements. Items were answered with “Reported”, “Not Reported”, or “Not Applicable”.

Three authors (QG, MP, and WT), blinded to each other's assessments, abstracted the reporting of each article independently. Instead of all three raters reviewing all articles, we decided to have two reviewers rate each article. To determine the number of articles needed to be evaluated by the second reviewer to ensure a desired level of reliability, we performed a sample size calculation [30], [31]. The sample size of 50 was chosen so as to estimate the kappa for the inter-rater agreement within a margin of error of 0.3 with 95% confidence, assuming that the true kappa would be 0.6 or more and that the proportion of agreements by chance was 0.7 or less (see File S2). The first reviewer (QG) evaluated all 100 articles, of which 50 articles were randomly selected for the second reviewer (MP), and the other 50 articles were given to the third reviewer (WT) for abstraction; each article was therefore rated by two reviewers.

After completion of independent assessments, any disagreements between any pair of reviewers (i.e., QG and MP; QG and WT) were resolved by discussion among two reviewers, and if necessary, involving the third reviewer or expert (GH) until consensus was reached. The raw data collected from the 100 studies is available at online Supporting Information (see File S4).

### Statistical Analysis

We calculated the percentage of studies that reported each evaluation item and a 95% confidence interval (CI) using an exact binomial method [32]. We then estimated the median, minimum and maximum percentages of reported items for each article.

Inter-rater agreement was assessed using the prevalence-adjusted bias-adjusted kappa (PABAκ) coefficient [33]. When the prevalence of a rating is very high or low, the value of kappa may indicate a low level of agreement while the observed percentage of agreement is high, known as the kappa paradox [34]. Hence, we used prevalence-adjusted bias-adjusted kappa [33] to address this paradox and to better interpret the inter-rater agreement. Kappa coefficient results were interpreted based on the scale as proposed by Byrt [35]: 0.00 or less (No agreement), 0.01–0.20 (Poor agreement), 0.21–0.40 (Slight agreement), 0.41–0.60 (Fair agreement), 0.61–0.80 (Good agreement), 0.81–0.92 (Very good agreement), 0.93–1.00 (Excellent agreement).

We performed a sample size calculation to determine the number of articles to be included in the extraction and analysis. A sample size of 100 was chosen so that with 95% confidence, we would be able to quantify the true percentage of articles that reported each item to within 10% (see File S1). All statistical analyses were conducted using the SAS 9.2 software (Cary, NC).

## Results

### Study Selection

After removing the duplicates, the initial search strategy identified 1120 unique articles. We screened the articles in a random order for eligibility until the quota of 100 eligible articles was reached. To reach this target, we assessed 1100 articles (see Figure S1 for a flow diagram). The list of the 100 eligible articles is included in File S3.

### Study Characteristics

Among the included 100 eligible articles published in six leading journals in 2010 and 2011, about 60% came from the journal NeuroImage. The majority of study designs were cross-sectional (94%). The funding source was reported in 78% of the citations, and came primarily from two or more different sources (77%) rather than from industry alone (1%). Fifty three percent of included articles were published in 2010 and the remaining forty seven percent in 2011. The median total number of subjects was 34 (first quartile (Q1)  = 26, third quartile (Q3)  = 48) ranging from 8 to 126, and most studies (79%) had a sample size of no more than 50 (see Table 1).

**Table 1:** Characteristics of Included fMRI Studies (Information Extracted from Each Article).

	All articles (n = 100)
Study Feature	Median (Q1, Q3) or %
Publication Journal	
Neuron	2
Nature Neuroscience	1
Proceedings of the National Academy ofSciences of the United States of America	4
Brain	22
Journal of Neuroscience	13
Neuroimage	58
Publication Year	
2010	53
2011	47
Study Design	
Case-control	0
Cohort	6
Cross-sectional	94
Number of Subjects	34 (26, 48)
Up to 10	2
10–50	77
51–100	17
More than 100	4
Funding Sources	
Completely funded by industry	1
Others	77
Not reported	22

Note: Q1 = first quartile or 25th percentile, Q3 = third quartile or 75th percentile.

### Items Commonly Reported

Of the 83 items, 22 items were reported by 85% or more of the 100 included articles. Specifically, all of the studies reported sample sizes. Most studies further described the manufacturer, field strength and model name of the scanner and the pulse sequence type (98%), statistical methods used for group modeling (97%), subjects' characteristics such as age and gender (94%), statistical methods used for within-subject modeling (92%), eligibility criteria on selecting subjects (91%), and whether statistical inferences were corrected for multiple comparisons (90%). Similarly, 86% of the articles reported how regions of interest (ROIs) were defined. Of 86 articles that reported analyses not conducted on the whole brain, 80 (93%) explained how regions were determined (see Tables 2–10).

**Table 2:** Percentage of articles reported each item, inter-rater agreement on the item and whether the item should be included in future shortened checklist relating to “Experimental Design”.

Item No	Description	% Reported	PABAκ	Item Selection*
		(95% CI)	(95% CI)	
1a	Described number of blocks, trials, experimental units per session or per subject	92 (84, 96)	0.90 (0.77, 0.97)	Included
1b	Stated length of each trial and interval between trials described	81 (71, 88)	0.76 (0.60, 0.87)	Included
1c#	If ISIs are variable, reported the mean and range of ISIs and how they were distributed (n = 39)	23 (11, 39)	0.76 (0.60, 0.87)	Included
1d#	If block designs, specified the length of blocks (n = 73)	79 (67, 87)	0.72 (0.55, 0.84)	Included
1e#	If event-related designs, stated whether the design was optimized for efficiency, and if so, stated how (n = 35)	22 (10, 40)	0.70 (0.53, 0.83)	Included
1f#	If mixed design, stated correlation between block and event regressors (n = 2)	50 (1, 98)	0.94 (0.83, 0.99)	Included
2a	Stated task instructions on what subjects were asked to do	92 (84, 96)	0.92 (0.80, 0.98)	Included
2b	Described what the Stimuli were and how many there were	69 (58, 77)	0.72 (0.55, 0.84)	Included
2c	Stated whether specific stimuli repeated across trials	49 (38, 59)	0.46 (0.26, 0.63)	Included
3	If the experiment had multiple conditions, stated what the specific planned comparisons were, or whether an omnibus ANOVA test was used	89 (81, 94)	0.90 (0.77, 0.97)	Included

Abbreviations: ISIs, inter-stimulus intervals; ANOVA, analysis of variance.

# The conditional item which is needed to report when the condition is met.

*To identify whether the item should be included in future shortened checklist. If excluded, the reasons for exclusion are given.

**Table 3:** Percentage of articles reported each item, inter-rater agreement on the item and whether the item should be included in future shortened checklist relating to “Study Subjects”.

Item No	Description	% Reported	PABAκ	Item Selection*
		(95% CI)	(95% CI)	
4a	Stated number of subjects	100 (96, 100)	1.00 (0.93, 1.00)	Included
4b	Stated age (mean and range)	92 (84, 96)	0.90 (0.77, 0.97)	Included
4c	Stated handedness	64 (53, 73)	0.98 (0.89, 0.99)	Included
4d	Stated number of males or females	95 (88, 98)	0.90 (0.77, 0.97)	Included
4e	Stated inclusion and exclusion criteria	91 (83, 95)	0.86 (0.72, 0.94)	Included
4f	If any subjects were scanned but then rejected from analysis after data collection, stated numbers and reasons for rejection	52 (41, 62)	0.82 (0.67, 0.92)	Included
4g#	For group comparisons, stated what variables (if any) were equated across groups (n = 90)	70 (59, 79)	0.56 (0.37, 0.71)	Included
5	Stated which IRB approved the protocol	94 (87, 97)	0.94 (0.83, 0.99)	Included
6	Stated how behavioral performance was measured (e.g., response time, accuracy)	56 (45, 65)	0.34 (0.14, 0.52)	Excluded due to much subjectivity and low inter-rater agreement. For example, some standard tools (e.g., E-Prime, Fiber-Optic-Button box) measure response timing and accuracy. If these tools are cited, is it safe to assume that the behavioral performance is measured? If not, what minimum details are required to report so as to score it as ‘reported’? Is this item required to report in every study? If not, under what condition?

Abbreviations: IRB, institutional review board.

# The conditional item which is needed to report when the condition is met.

*To identify whether the item should be included in future shortened checklist. If excluded, the reasons for exclusion are given.

**Table 4:** Percentage of articles reported each item, inter-rater agreement on the item and whether the item should be included in future shortened checklist relating to “Image Properties”.

Item No	Description	% Reported	PABAκ	Item Selection*
		(95% CI)	(95% CI)	
7a	Provided manufacturer, field strength (in Tesla) and model name of MRI system	98 (92, 99)	0.96 (0.86, 0.99)	Included
7b	Gave number of experimental sessions and volumes acquired per session	50 (39, 60)	0.78 (0.62, 0.88)	Included
7c	Stated pulse sequence type (e.g., gradient/spin echo, EPI/spiral)	98 (92, 99)	1.00 (0.93, 1.00)	Included
7d	Stated field of view, matrix size, slice thickness, inter-slice skip	36 (26, 46)	0.76 (0.60, 0.87)	Included
7e	Provided acquisition orientation (axial, sagittal, coronal, oblique)	71 (61, 79)	0.90 (0.77, 0.97)	Included
7f	Stated whether it is on the whole brain. If not, state area of acquisition	65 (54, 74)	0.90 (0.77, 0.97)	Included
7g	Stated order of acquisition of slices (sequential or interleaved)	21 (13, 30)	0.82 (0.67, 0.92)	Included
7h	Stated TE, TR and flip angle	86 (77, 92)	0.92 (0.80, 0.98)	Included

Abbreviations: EPI, Echo Planar Imaging; TE, echo time; TR, repetition time.

*To identify whether the item should be included in future shortened checklist. If excluded, the reasons for exclusion are given.

**Table 5:** Percentage of articles reported each item, inter-rater agreement on the item and whether the item should be included in future shortened checklist relating to “Data Preprocessing”.

Item No	Description	% Reported	PABAκ	Item Selection*
		(95% CI)	(95% CI)	
8a	Stated the version number or date of last application for each piece of software used	78 (68, 85)	0.76 (0.60, 0.87)	Included
8b	Specified differences in any subjects who required different processing operations or settings in the analysis (n = 78)	3 (1, 10)	0.60 (0.42, 0.75)	Excluded due to much subjectivity. For example, if the study states that all subjects received same operations or settings, this item would not be applicable. If there is no indication of this, it is difficult to decide under what condition this item is expected to be reported.
9a	Specified order of preprocessing operations	26 (17, 35)	0.70 (0.53, 0.83)	Included
9b	Stated reference slice and interpolation type for slice timing correction	9 (4, 16)	0.94 (0.83, 0.99)	Included
9c	Stated reference scan, image similarity metric, type of interpolation used, degrees-of-freedom, and ideally optimization method for motion correction	15 (8, 23)	0.74 (0.58, 0.86)	Included

*To identify whether the item should be included in future shortened checklist. If excluded, the reasons for exclusion are given.

**Table 6:** Percentage of articles reported each item, inter-rater agreement on the item and whether the item should be included in future shortened checklist relating to “Inter-subject Registration and Smoothing”.

Item No	Description	% Reported	PABAκ	Item Selection*
		(95% CI)	(95% CI)	
10a	Illustrated the voxels presented in all subjects using “mask image”	16 (9, 24)	0.68 (0.51, 0.81)	Included
10b	Described transformation model (linear/affine, nonlinear), type of any non-linear transformations (polynomial, discrete cosine basis), number of parameters (e.g., 12 parameter affine), regularization image-similarity metric, and interpolation method	18 (11, 26)	0.70 (0.53, 0.83)	Included
10c	Stated object anatomical image information used for transformation to Atlas	42 (32, 52)	0.46 (0.26, 0.63)	Included
10d	Stated if anatomical MRI is co-planar with functional acquisition	36 (26, 46)	0.80 (0.65, 0.90)	Included
10e	Stated if functional acquisition is co-registered to anatomical	47 (36, 57)	0.82 (0.67, 0.92)	Included
10f#	If functional acquisition is co-registered to anatomical, stated how (n = 47)	27 (15, 42)	0.50 (0.31, 0.66)	Included
10g	Provided Atlas/target information	87 (78, 92)	0.66 (0.48, 0.79)	Included
10h	Stated brain image template space, name, modality and resolution (e.g., “FSL's MNI Avg152, T1 2×2×2 mm”, “SPM2's MNI gray matter template 2×2×2 mm”)	16 (9, 24)	0.64 (0.46, 0.78)	Included
10i	Stated typically MNI, Talairach, or MNI converted to Talairach	85 (76, 91)	0.84 (0.69, 0.93)	Included
10j#	If MNI is converted to Talairach, stated the method used (e.g., Brett's mni2tal) (n = 13)	61 (31, 86)	0.86 (0.72, 0.94)	Included
10k	State clearly how anatomical locations (e.g., gyral anatomy, Brodmann areas) were determined (e.g., paper atlas, Talairach Daemon, manual inspection of individual's anatomy, etc.)	61 (50, 70)	0.68 (0.50, 0.81)	Included
11	Described size and type of smoothing kernel (e.g., for a group study, “12 mm FHWM Gaussian smoothing applied to ameliorate differences in inter-subject localization”; for single subject fMRI “6 mm FWHM Gaussian smoothing used to reduce noise”)	84 (75, 90)	0.96 (0.85, 0.99)	Included

Abbreviations: MRI, magnetic resonance imaging; MNI, Montreal Neurological Institute space.

# The conditional item which is needed to report when the condition is met.

*To identify whether the item should be included in future shortened checklist. If excluded, the reasons for exclusion are given.

**Table 7:** Percentage of articles reported each item, inter-rater agreement on the item and whether the item should be included in future shortened checklist relating to “Statistical Modeling”.

Item No	Description	% Reported	PABAκ	Item Selection*
		(95% CI)	(95% CI)	
12	For novel methods not described in a separate paper, provided description and validation of method in the text or an appendix (n = 2)	50 (1, 98)	0.88 (0.74, 0.96)	Excluded. Given that methods are continually developing, it involves much subjectivity as to whether or not the reported methods are novel.
13a	Stated statistical model and estimation method for both intra-subject and group modeling described	92 (84, 96)	0.80 (0.65, 0.90)	Included
13b	Stated block- or epoch-based or event-related model	97 (91, 99)	0.92 (0.80, 0.98)	Included
13c	Specified hemodynamic response function	58 (47, 67)	0.76 (0.60, 0.87)	Included
13d	Clearly stated additional regressors used (e.g., temporal derivatives, motion, behavioral covariates)	53 (42, 63)	0.58 (0.39, 0.73)	Included
13e	Stated any orthogonalization of regressors	7 (2, 13)	0.86 (0.72, 0.94)	Included
13f	Stated drift modeling or high-pass filtering (e.g., “DCT with cut off of X seconds”; “Gaussian-weighted running line smoother, cut-off 100 seconds”, or “cubic polynomial”)	55 (44, 64)	0.74 (0.57, 0.86)	Included
13g	Described autocorrelation model (e.g., AR(1), AR(1)+WN, or arbitrary autocorrelation function)	18 (11, 26)	0.80 (0.64, 0.90)	Included
13h	Defined contrast for task or stimulus conditions	90 (82, 95)	0.90 (0.77, 0.97)	Included
14a	Stated statistical model, estimation method and inference type for group modeling (e.g., mixed, random or fixed effects)	97 (91, 99)	0.90 (0.77, 0.97)	Included
14b#	If fixed effects inference used for group modeling, provided the justification (n = 31)	3 (1, 16)	0.46 (0.26, 0.63)	Included
14c	If the group has more than 2-levels, described the levels and assumptions of the model (e.g., are variances assumed equal between groups) (n = 21)	28 (11, 52)	0.60 (0.41, 0.75)	Included
14d	Stated methods used for repeated measures to account for within subject correlation in group modeling	24 (16, 33)	0.66 (0.48, 0.79)	Included

Abbreviations: DCT, discrete cosine transform; AR(1), first-order Autoregressive Model; WN, white noise.

# The conditional item which is needed to report when the condition is met.

*To identify whether the item should be included in future shortened checklist. If excluded, the reasons for exclusion are given.

**Table 8:** Percentage of articles reported each item, inter-rater agreement on the item and whether the item should be included in future shortened checklist relating to “Statistical Inference on Statistic Image (thresholding)”.

Item No	Description	% Reported	PABAκ	Item Selection*
		(95% CI)	(95% CI)	
15a	Stated type of search region for analysis, and the volume in voxels or CC	54 (43, 64)	0.60 (0.41, 0.75)	Included
15b#	If not whole brain, stated how region was determined (n = 86)	93 (85, 97)	0.58 (0.39, 0.73)	Included
15c#	Stated and listed each if threshold used for inference and threshold used for visualization in figures is different (n = 49)	44 (30, 59)	0.56 (0.37, 0.71)	Included
15d	Stated if inferences are corrected for multiple comparisons	90 (82, 95)	0.80 (0.64, 0.90)	Included
15e#	If correction is limited to a small volume, stated the method for selecting the region (n = 73)	72 (60, 82)	0.54 (0.35, 0.70)	Included
15f#	Labeled “uncorrected” if no formal multiple comparisons method is used (n = 76)	84 (74, 91)	0.80 (0.64, 0.90)	Included
15g	Stated if it is voxel-wise significance	49 (38, 59)	0.54 (0.35, 0.70)	Included
15h	Stated if inferences are corrected for FWE or FDR	50 (39, 60)	0.78 (0.62, 0.89)	Included
15i#	Listed the smoothness in mm FWHM and the RESEL count if FWE found by random field theory (n = 45)	25 (1, 80)	0.70 (0.52, 0.83)	Included
15j#	Provided details of parameters for simulation if FWE found by simulation (e.g., AFNI AphaSim) (n = 7)	57 (18, 90)	0.62 (0.43, 0.76)	Included
15k#	If not a standard method, specified the method for finding significance (n = 12)	100 (73, 100)	0.72 (0.55, 0.84)	Included
15l	Stated cluster-defining threshold (e.g., *P* = *0.001*)	51 (40, 61)	0.44 (0.24, 0.61)	Included
15m	Stated the corrected cluster significance level (e.g., “Statistic images were assessed for cluster-wise significance using a cluster-defining threshold of *P* = 0.001; the 0.05 FWE-corrected critical cluster size was 103”)	55 (44, 64)	0.42 (0.22, 0.59)	Included
15n#	Provided smoothness and RESEL count if significance determined with random field theory (n = 8)	12 (1, 52)	0.96 (0.85, 0.99)	Included
15o	Stated correction for multiple planned comparisons based upon each voxel	14 (7, 22)	0.44 (0.24, 0.61)	Included
15p#	Stated observed effect size for any failure to reject the null hypothesis (e.g., lack of activation in a particular region) (n = 1)	0 (0, 3)	0.98 (0.89, 0.99)	Included

Abbreviations: CC, cubic centimeter; FWE, family-wise error; FDR, false discovery rate; FWHM, full-width at half-maximum; RESEL, resolution element.

# The conditional item which is needed to report when the condition is met.

*To identify whether the item should be included in future shortened checklist. If excluded, the reasons for exclusion are given.

**Table 9:** Percentage of articles reported each item, inter-rater agreement on the item and whether the item should be included in future shortened checklist relating to “Statistical Inference on ROI Analysis”.

Item No	Description	% Reported	PABAκ	Item Selection*
		(95% CI)	(95% CI)	
16a	Described how ROIs were defined (e.g., functional or anatomical localizer)	86 (77, 92)	0.54 (0.35, 0.70)	Included
16b	Described how signal was extracted within ROI (e.g., average parameter estimates, FIR deconvolution)	45 (35, 55)	0.46 (0.26, 0.63)	Included
16c#	If percent signal change reported, described how scaling factor was determined (n = 35)	34 (19, 52)	0.52 (0.32, 0.68)	Included
16d	Stated if percent signal change is relative to voxel-mean, or whole-brain mean	16 (9, 24)	0.66 (0.48, 0.79)	Included

Abbreviations: ROI, region of interest; FIR, finite impulse response.

# The conditional item which is needed to report when the condition is met.

*To identify whether the item should be included in future shortened checklist. If excluded, the reasons for exclusion are given.

**Table 10:** Percentage of articles reported each item, inter-rater agreement on the item and whether the item should be included in future shortened checklist relating to “Figures and Tables”.

Item No	Description	% Reported	PABAκ	Item Selection*
		(95% CI)	(95% CI)	
17a	Stated the statistical map that the figure or table is based upon (e.g., *Z, t, p*)	95 (88, 98)	0.84 (0.69, 0.93)	Included
17b	Provided the thresholds used to create the image or figure (e.g., intensity and cluster extent)	71 (61, 79)	0.60 (0.41, 0.75)	Included
18	Underlying anatomical image stated (e.g., average anatomy, template image)	26 (17, 35)	0.66 (0.48, 0.79)	Included
19a	Locations in stereotactic space provided	73 (63, 81)	0.80 (0.64, 0.90)	Included
19b	Provided statistics for each cluster including maximum and cluster extent	51 (40, 61)	0.86 (0.72, 0.94)	Included
19c	Provided source of anatomical labels (e.g., atlas, automated labeling method)	67 (56, 76)	0.62 (0.43, 0.76)	Included

*To identify whether the item should be included in future shortened checklist. If excluded, the

### Items Not Commonly Reported

Among the 83 items, a total of 31 items were reported by no more than 50% of the included articles; 13 items were reported by fewer than 20% of the articles. Critically, and in sharp contrast to Poldrack's guidelines, none of the studies reported observed effect sizes if they failed to reject the null hypothesis. Only one article (3%, 1/31) provided justifications for using fixed-effect inferences for group modeling. Other items that were insufficiently reported included slice-timing and motion corrections (12/100), temporal autocorrelation modeling used to account for within-subject variances and correlations (18/100), whether and how the task design was optimized for efficiency if it was an event-related design (22%, 8/35), distributions of inter-stimulus intervals (ISI), whether ISI was variable (23%, 9/39), statistical methods for repeated measurements (24/100), and smoothness and resolution element (RESEL) count if family-wise error (FWE) was found by random field theory (RFT) (25%, 1/4). Moreover, only six articles (28%, 6/21) described whether variances were assumed equal among groups if there were more than two groups. Of the 35 articles that reported percent signal changes, 12 (34%, 12/35) explained how scaling factors were determined. Similarly, 45% (45/100) of the articles stated how signal was extracted within ROIs.

### Reported Items per Article

The median (minimum, maximum) percentage of reported items per article was 51% (30%, 78%).

The inter-rater agreement was very good (PABAκ >0.8) for 31 items, good (0.6< PABAκ ≤0.8) for 31 items, fair (0.4<PABAκ ≤0.6) for 20 items, and slight (PABAκ = 0.34) for one item (Table 2–10). We note that some items had a lower inter-rater agreement than the others. This may be due to varying interpretations of items among reviewers. For example, item 6 (“State how behavioral performance was measured”) had the lowest kappa statistic because it involved much subjectivity (e.g., if standard tools including E-Prime were cited, was it safe to assume the item was reported? Or if not the standard tool, what minimum details should be reported? Was this item necessary to report in each study?). We used duplicate reviewers and the consensus among reviewers to help reduce the biases and hence increase the reliability of findings.

### Specifics on Reported Items

Manuscript quality hinges not only on whether an item was reported, but the specifics of the method that was used. Here we describe manuscripts' methodological choices regarding software, spatial smoothing, temporal filtering and thresholding for statistical significance.

Seventy-eight percent of the articles reported a version of the software package used in fMRI data analyses (see Table 5), and 98% reported using at least one software package. Of the 98 articles, 71.4% used SPM, 11.2% used FSL, and 10.2% used BrainVoyager (Table 11). The packages used by fewer than 10 articles include AFNI (7.1%), MATLAB (6.1%) and XBAM (1.0%). Many software packages were reported with a version; SPM5 was the most commonly used by 43.9% (43/98) of the articles, followed by SPM2 (17.3%, 17/98), SPM8 (8.2%, 8/98), and FSL-no version (6.1, 6/98). No version of XBAM was specified (see Table 11 for details).

**Table 11:** The use of software packages and versions.

	Reporting Articles	(N = 98)
Type of Software	Frequency	%
AFNI (no version)	7	7.1
BrainVoyager	10	10.2
* BrainVoyager2.1*	1	1.0
* BrainVoyager2000*	1	1.0
* BrainVoyagerQX1.10.4*	1	1.0
* BrainVoyagerQX1.9*	1	1.0
* BrainVoyagerQX2*	1	1.0
* BrainVoyagerQX (no version)*	3	3.1
* BrainVoyager (no version)*	2	2.1
FSL	11	11.2
* FSL3.3*	2	2.1
* FSL4.1*	1	1.0
* FSL4.1.4*	1	1.0
* FSL5.9.2*	1	1.0
* FSL (no version)*	6	6.1
MATLAB	6	6.1
* MATLAB6*	1	1.0
* MATLAB6.5*	1	1.0
* MATLAB7.2*	1	1.0
* MATLAB (no version)*	3	3.1
SPM	70	71.4
* SPM2*	17	17.3
* SPM5*	43	43.9
* SPM8*	8	8.2
* SPM99*	1	1.0
* SPM (no version)*	1	1.0
XBAM (no version)	1	1.0

Abbreviations: AFNI, Analysis of Functional NeuroImages; FSL, FMRIB Software Library; SPM, Statistical Parametric Mapping; XBAM, Brain Activation Mapping.

reasons for exclusion are given.

Spatial smoothing reduces noise and hence increases the signal-to-noise ratio while reducing the resolution of data [36], [37]. Therefore, it is important to specify the extent to which spatial smoothing that has been applied. Specifically, the size of the smoothing kernel determines how much the data is smoothed, which has an effect on the extent of within-subject variability of estimates [38]. Reporting smoothing parameters helps readers to determine the balance between improving the sensitivity and maintaining the resolution of the functional image. As can be seen in Table 12, the majority of studies reported using spatial smoothing (88/100), with 95.5% (84/88) specifying a type of kernel. The widths of smoothing kernel ranged from 3 mm to 12 mm with a median width of 8 mm. The most frequent kernel width was 8 mm (42%, 37/88). Other common widths included 6 mm (29.5%, 26/88), 9 mm (8%, 7/88), and 10 mm (5.7%, 5/88). The widths used by fewer than 5 studies were 5 mm, 12 mm, 4 mm, 4.2 mm and 3 mm. None of the studies justified their choices of smoothing kernel.

**Table 12:** The use of spatial smoothing, temporal filtering, and between-subject inference.

	Reporting Articles	
Parameter	Frequency	%
**Spatial Smoothing**		
Use of Spatial Smoothing (N = 100)	88	88
Type of Kernel (N = 88)	84	95.5
Width of Smoothing Kernel (FWHM, N = 88)		
*8* *mm*	37	42.0
*6* *mm*	26	29.5
*9* *mm*	7	8.0
*10* *mm*	5	5.7
*5* *mm*	4	4.5
*12* *mm*	3	3.4
*4* *mm*	2	2.3
*4.2* *mm*	1	1.1
*3* *mm*	1	1.1
*Median (min, max)*	8 mm (3 mm, 12 mm)	
Justification for the Chosen Smoothing Kernel	0	0
**Temporal Filtering**		
Use of Temporal Filtering (N = 100)	61	61
Type of Filtering (N = 60)		
*High-pass*	57	95
*Low-pass*	1	1.7
*Band-pass*	2	3.3
Filter Cut-off (second)		
*High-pass: Median (min, max)*	128 s (2.8 s, 318 s)	
*Low-pass: Median (min, max)*	6.7 s (6.7 s, 6.7 s)	
**Between-subject Inference**		
Use of Per-voxel (height) Threshold (N = 100)	78	78
Size of Per-voxel Threshold (N = 78)		
*p<0.001*	25	32.1
*p<0.05*	24	30.8
*p<0.01*	13	16.7
*p<0.005*	12	15.4
*Others*	11	14.1
Use of Cluster-extent Threshold (N = 100)	63	63
Size of Cluster-extent Threshold (mm^3^)		
*Median (min, max)*	184 (3, 5625)	
Use of Formal Corrections for Multiple Comparison	81	81
Methods Used for Formal Corrections (N = 81)		
*Family-Wise Error*	23	28.4
*False Discovery Rate*	22	27.2
*Monte Carlo Simulation*	15	18.5
*Gaussian Random Field Theory*	4	4.9
*Other Methods*	4	4.9
*Not Reported*	13	16.1

Abbreviation: FWHM, Full Width at Half Maximum.

As with spatial smoothing, temporal filtering aims to increase the signal-to-noise ratio. Since most of the noise in fMRI is low frequency, high-pass filtering improves the ratio better than low-pass filtering, and is almost as good as band-pass filtering [36], [39]. Specifying the filter cut-off parameter helps understand the temporal filtering process. Most studies (61/100) reported whether temporal filtering was used. Of the 60 studies that reported actual use of temporal filtering, most (95%, 57/60) used high-pass filtering. Only a few studies used low-pass (1.7%, 1/60) and band-pass (3.3%, 2/60) temporal filtering. Forty-eight studies reported the filter cut-off, among which the high-pass filtering cut-off ranged from 2.8 s to 318 s with a median and mode value of 128 s, compared to low-pass filtering with a single cut-off value of 6.7 s.

The threshold for statistical significance in voxel- or cluster-level analysis controls the type I error rate [40], and many papers have suggested using formal correction methods [40]–[45]. Of the 100 included studies, 78% reported the use of per-voxel (or height) threshold. The most common per-voxel threshold was *p*<0.001 (32.1%, 25/78), followed by *p*<0.05 (30.8%, 24/78), *p*<0.01 (16.7%, 13/78), and *p*<0.005 (15.4%, 12/78). More than half of the studies (63/100) reported using cluster-extent threshold. The size of cluster-extent threshold ranged from 3 mm^3^ to 5625 mm^3^ with a median threshold of 184 mm^3^. The majority of studies (81%, 81/100) reported using corrections for multiple testing; among these studies, around 16.1% (13/81) did not report which correction method was used. Among the studies that reported a method, the correction methods included False-wise Error (28.4%, 23/81), False Discovery Rate (27.2%, 22/81), Monte Carlo Simulation (18.5%, 15/81), Gaussian Random Field Theory (4.9%, 4/81) and several others (4.9%, 4/81).

## Discussion

This study identified some reporting practices in observational clinical fMRI studies that met expectations and other areas where reporting was less than adequate. In particular, only one quarter of the items from the recommended reporting guidelines by Poldrack et al. (2008) were reported adequately. Indeed, only one half of recommended items were routinely reported in each article. Moreover, one third of the items were reported by less than half of the articles. Less adequately reported items were distributed across the categories: experimental design, inter-subject registration and smoothing, data preprocessing, statistical modeling, and statistical inference on ROI analysis. These results indicate that substantial room for improvement exists in the reporting of observational clinical fMRI studies.

Specifically, improvement in reporting important details is recommended in areas such as observed effect sizes in the results section when study results are negative, justifications for fixed-effect inferences used for group modeling, and temporal autocorrelation matrix used to account for within-subject variance and correlations. As effect sizes observed from statistically significant regions overestimate true effect sizes [46], [47], including values from non-significant regions (e.g., those that are identified from similar previous studies) would help provide a more realistic range of effect size estimates and reduce the risk of bias arising from reporting on active regions only. Given the existence of temporal autocorrelation in fMRI time series, incorporating an autocorrelation structure increases the accuracy of variance estimates. Reporting temporal autocorrelation estimates enables proper power analyses based on the method proposed by Mumford and Nichols [48]. Whereas findings from fixed-effect inferences particularly reflect the cohort of subjects studied, random-effect inferences generalize findings to the population at large from which the study sample was drawn [49]. The current recommendation is to use random-effect inferences for between-subject group modeling and fixed-effect inferences for single-subject modeling. Providing justifications for using fixed-effects for group modeling would enhance understanding and interpretation.

This study differed substantially from the one existing review of fMRI reporting [18] in the number of items, definitions of items, study population and study design. For example, although Carp's study used a single reviewer, we conducted a systematic review by using a duplicate abstraction, measuring inter-rater agreement and resolving disagreements through consensus. Moreover, our study focused on observational studies with clinical participants; in contrast, Carp evaluated fMRI studies in general which may not capture many studies involving clinical participants. There are also some notable differences in results between the two studies. For example, in the current study around one-third reported the distribution of inter-trial intervals, compared to one-twelfth in Carp's study. About one half reported the number of subjects rejected from analyses with reasons for rejection in our study, which is one quarter greater than that of Carp's study. Similarly, less than one-third of the articles in our study reported the following four methodological items but still showed better reporting than those in Carp's study: how potentially confounding variables were matched across groups for group comparisons, whether autocorrelations were modeled, whether equal variance was assumed across groups for multiple group designs, and the number of RESELs and image smoothness for studies using FWE correction. Unfortunately, we are unable to identify the specific factors associated with these differences between the current study and Carp's study; the factors might be the type of clinical participants involved in the study, impact factors of the journal, or the exclusion of studies of connectivity. Future research may be helpful in this regard by comparing reporting quality among studies with clinical participants versus without clinical participants, with high impact factor journals versus with low impact factor journals, and including studies of connectivity versus excluding connectivity. Although different, both studies did detect some commonality in important items that are frequently absent from published reports, indicating that incomplete reporting challenges the evaluation, understanding and interpretation of study findings, and limits the use of results for synthesis, e.g., for meta analyses.

Complete reporting becomes particularly important for studies involving clinical populations, where ensuring methodological rigor is necessary to uphold investigators' promises to their participants that their participation will help society to better understand the nature of their condition. Our findings point towards the need for substantial improvement in this regard. In several other fields of health research, it has been demonstrated that journals adopting standard reporting guidelines (e.g., CONSORT statement) have better quality of reporting than those that do not [50]–[52], thus the use of guidelines in the fMRI literature may help improve the quality of reporting as well.

Implementation of the guidelines for reporting fMRI studies proposed by Poldrack and his colleagues (2008) do face some challenges. Firstly, authors often have strict word limits and the current guidelines are lengthy, making it important to identify which items are most essential. Secondly, some items are relevant to the quality of reporting observational clinical studies but are not covered in Poldrack et al.'s guidelines (for example, sample size calculations in the methods section, characteristics of clinical participants, and participation data flow diagrams to better understand potential bias due to non-participation [53]). Since reporting guidelines are evolving documents [54], we suggest dividing the list of items that should be reported into those that are essential, which should be placed in the manuscript itself, and those which are helpful to report can be included as online supplements. Some methodological parameters have more impact than others [28], [55] and hence should be considered as essential items. Some journals (e.g., Nature) have recently removed space limitations on methods sections, however, since this is not a widespread practice it would still be useful to distinguish between essential and helpful items. In addition to the form of text-based reporting, some items can be reported in the form of source code (e.g., for data collection and statistical analyses) [56] and machine-readable information compatible to different imaging analyses packages [57]. Our recommendation for creating a list of essential items is not intended to supplant the existing guidelines but rather a suggestion to consider during the next update of the guidelines. We hope that our suggestions will lead to more discussion and future consensus regarding what is in fact essential to report in the manuscript itself for observational clinical fMRI studies. For example, the consensus can be reached through a consensus meeting involving a variety of experts in this area, in a similar way that the standard CONSORT guideline was created. Involving journal editors in the process and having their endorsement of the guidelines would encourage researchers to comply with the new standards.

The present study has several limitations. First, findings in this study reflect the quality of reporting of observational clinical fMRI studies in six top neuroscience journals published between 2010 and 2011, results that may not apply to journals in general. Most likely, these results may overestimate true rates of reporting. Second, several items on the checklist used for evaluation in this systematic review involve subjectivity. However, using duplicate review and consensus for any disagreements helped to reduce differences in interpretations between reviewers.

## Conclusion

This study has highlighted under-reported areas in observational fMRI studies involving clinical participants and points towards a need for improvement. Adherence to the guidelines for fMRI studies proposed by Poldrack and his colleagues could help improve quality of reporting. Considering that the guidelines are evolving and need continual updates, we suggest constructing a checklist that captures essential items to report to accommodate practical needs, and enforcing the reporting guidelines through proposed ways.

## Supporting Information

Figure S1 **Flow Diagram of Citation Selection Process.**
(DOC)

Checklist S1 **PRISMA 2009 Checklist.**
(DOC)

File S1 **Sample size calculation for estimating a single proportion with a level of confidence.**
(DOC)

File S2 **Sample size calculation for estimating a Cohen's kappa coefficient with a given precision.**
(DOC)

File S3 **List of 100 eligible studies.**
(DOC)

File S4 **Raw data collected from the 100 studies.**
(XLS)

Table S1 **Search strategy for Ovid Medline database.**
(DOC)

Table S2 **Data extraction form containing 83 items adapted from Poldrack et al.'s checklist.**
(DOC)

## References

[1] Huettel SA, Song AW, McCarthy G (2009) Functional magnetic resonance imaging: Sunderland, MA: Sinauer Associates, Inc.

[2] Carter CS, Heckers S, Nichols T, Pine DS, Strother S (2008) Optimizing the design and analysis of clinical functional magnetic resonance imaging research studies. Biol Psychiatry 64: 842–849. 18718572 10.1016/j.biopsych.2008.06.014

[3] Sheline YI, Barch DM, Donnelly JM, Ollinger JM, Snyder AZ, et al (2001) Increased amygdala response to masked emotional faces in depressed subjects resolves with antidepressant treatment: An fMRI study. Biol Psychiatry 50: 651–658. 11704071 10.1016/s0006-3223(01)01263-x

[4] Siegle GJ, Steinhauer SR, Thase ME, Stenger VA, Carter CS (2002) Can't shake that feeling: Event-related fMRI assessment of sustained amygdala activity in response to emotional information in depressed individuals. Biol Psychiatry 51: 693–707. 11983183 10.1016/s0006-3223(02)01314-8

[5] Glahn DC, Ragland JD, Abramoff A, Barrett J, Laird AR, et al (2005) Beyond hypofrontality: A quantitative meta-analysis of functional neuroimaging studies of working memory in schizophrenia. Hum Brain Mapp 25: 60–69. 15846819 10.1002/hbm.20138 PMC6871703

[6] Snitz BE, MacDonald A III, Cohen JD, Cho RY, Becker T, et al (2005) Lateral and medial hypofrontality in first-episode schizophrenia: Functional activity in a medication-naive state and effects of short-term atypical antipsychotic treatment. Am J Psychiatry 162: 2322–2329. 16330597 10.1176/appi.ajp.162.12.2322

[7] Monk CS, Klein RG, Telzer EH, Schroth EA, Mannuzza S, et al (2008) Amygdala and nucleus accumbens activation to emotional facial expressions in children and adolescents at risk for major depression. Am J Psychiatry 165: 90–98. 17986682 10.1176/appi.ajp.2007.06111917

[8] Yoon JH, Minzenberg MJ, Ursu S, Ryan Walter BS, Wendelken C, et al (2008) Association of dorsolateral prefrontal cortex dysfunction with disrupted coordinated brain activity in schizophrenia: Relationship with impaired cognition, behavioral disorganization, and global function. Am J Psychiatry 165: 1006–1014. 18519527 10.1176/appi.ajp.2008.07060945 PMC3985422

[9] von Elm E, Altman DG, Egger M, Pocock SJ, Gotzsche PC, et al (2007) The strengthening the reporting of observational studies in epidemiology (STROBE) statement: Guidelines for reporting observational studies. Ann Intern Med 147: 573–577. 17938396 10.7326/0003-4819-147-8-200710160-00010

[10] Begg C, Cho M, Eastwood S, Horton R, Moher D, et al (1996) Improving the quality of reporting of randomized controlled trials. the CONSORT statement. JAMA 276: 637–639. 8773637 10.1001/jama.276.8.637

[11] Chan AW, Krleza-Jeric K, Schmid I, Altman DG (2004) Outcome reporting bias in randomized trials funded by the canadian institutes of health research. CMAJ 171: 735–740. 15451835 10.1503/cmaj.1041086 PMC517858

[12] Chan AW, Altman DG (2005) Identifying outcome reporting bias in randomised trials on PubMed: Review of publications and survey of authors. BMJ 330: 753. 15681569 10.1136/bmj.38356.424606.8F PMC555875

[13] Dwan K, Altman DG, Arnaiz JA, Bloom J, Chan AW, et al (2008) Systematic review of the empirical evidence of study publication bias and outcome reporting bias. PLoS One 3: e3081. 18769481 10.1371/journal.pone.0003081 PMC2518111

[14] Poldrack RA, Fletcher PC, Henson RN, Worsley KJ, Brett M, et al (2008) Guidelines for reporting an fMRI study. Neuroimage 40: 409–414. 18191585 10.1016/j.neuroimage.2007.11.048 PMC2287206

[15] Young NS, Ioannidis JP, Al-Ubaydli O (2008) Why current publication practices may distort science. PLoS Med 5: e201. 18844432 10.1371/journal.pmed.0050201 PMC2561077

[16] Langan S, Schmitt J, Coenraads PJ, Svensson A, von Elm E, et al (2010) The reporting of observational research studies in dermatology journals: A literature-based study. Arch Dermatol 146: 534–541. 20479302 10.1001/archdermatol.2010.87

[17] Papathanasiou AA, Zintzaras E (2010) Assessing the quality of reporting of observational studies in cancer. Ann Epidemiol 20: 67–73. 20006277 10.1016/j.annepidem.2009.09.007

[18] Carp J (2012) The secret lives of experiments: Methods reporting in the fMRI literature. Neuroimage 63: 289–300. 22796459 10.1016/j.neuroimage.2012.07.004

[19] Guo Q, Thabane L, Hall G, McKinnon M, Goeree R, et al (2014) A systematic review of the reporting of sample size calculations and corresponding data components in observational functional magnetic resonance imaging studies. Neuroimage 86: 172–181. 23954487 10.1016/j.neuroimage.2013.08.012

[20] MacDonald AW III, Thermenos HW, Barch DM, Seidman LJ (2009) Imaging genetic liability to schizophrenia: Systematic review of FMRI studies of patients' nonpsychotic relatives. Schizophr Bull 35: 1142–1162. 18556667 10.1093/schbul/sbn053 PMC2762618

[21] Huang W, Pach D, Napadow V, Park K, Long X, et al (2012) Characterizing acupuncture stimuli using brain imaging with FMRI—a systematic review and meta-analysis of the literature. PLoS One 7: e32960. 22496739 10.1371/journal.pone.0032960 PMC3322129

[22] Lee KP, Schotland M, Bacchetti P, Bero LA (2002) Association of journal quality indicators with methodological quality of clinical research articles. JAMA 287: 2805–2808. 12038918 10.1001/jama.287.21.2805

[23] Birken CS, Parkin PC (1999) In which journals will pediatricians find the best evidence for clinical practice? Pediatrics 103: 941–947. 10224169 10.1542/peds.103.5.941

[24] Opthof T (1997) Sense and nonsense about the impact factor. Cardiovasc Res 33: 1–7. 9059521 10.1016/s0008-6363(96)00215-5

[25] Schoonbaert D, Roelants G (1996) Citation analysis for measuring the value of scientific publications: Quality assessment tool or comedy of errors? Trop Med Int Health 1: 739–752. 8980585 10.1111/j.1365-3156.1996.tb00106.x

[26] Bruer JT (1982) Methodological rigor and citation frequency in patient compliance literature. Am J Public Health 72: 1119–1123. 7114334 10.2105/ajph.72.10.1119 PMC1650176

[27] Lazar NA (2008) The statistical analysis of functional MRI data. New York, NY: Springer-Verlag New York.

[28] Strother S, La Conte S, Kai Hansen L, Anderson J, Zhang J, et al (2004) Optimizing the fMRI data-processing pipeline using prediction and reproducibility performance metrics: I. A preliminary group analysis. Neuroimage 23 Supplement 1S196–S207. 15501090 10.1016/j.neuroimage.2004.07.022

[29] Churchill NW, Oder A, Abdi H, Tam F, Lee W, et al (2012) Optimizing preprocessing and analysis pipelines for single-subject fMRI. I. standard temporal motion and physiological noise correction methods. Hum Brain Mapp 33: 609–627. 21455942 10.1002/hbm.21238 PMC4898950

[30] Altman DG (1991) Practical statistics for medical research. Chapman and Hall/CRC.

[31] El Emam K, Jonker E, Arbuckle L, Malin B (2011) A systematic review of re-identification attacks on health data. 12 (6). 10.1371/journal.pone.0028071 PMC3229505 22164229

[32] Clopper C, Pearson ES (1934) The use of confidence or fiducial limits illustrated in the case of the binomial. Biometrika 26: 404–413.

[33] Byrt T, Bishop J, Carlin JB (1993) Bias, prevalence and kappa. J Clin Epidemiol 46: 423–429. 8501467 10.1016/0895-4356(93)90018-v

[34] Feinstein AR, Cicchetti DV (1990) High agreement but low kappa: I. the problems of two paradoxes. J Clin Epidemiol 43: 543–549. 2348207 10.1016/0895-4356(90)90158-l

[35] Byrt T (1996) How good is that agreement? Epidemiology 7: 561. 10.1097/00001648-199609000-00030 8862998

[36] Skudlarski P, Constable RT, Gore JC (1999) ROC analysis of statistical methods used in functional MRI: Individual subjects. Neuroimage 9: 311–329. 10075901 10.1006/nimg.1999.0402

[37] Hopfinger JB, Buchel C, Holmes AP, Friston KJ (2000) A study of analysis parameters that influence the sensitivity of event-related fMRI analyses. Neuroimage 11: 326–333. 10725188 10.1006/nimg.2000.0549

[38] Desmond JE, Glover GH (2002) Estimating sample size in functional MRI (fMRI) neuroimaging studies: Statistical power analyses. J Neurosci Methods 118: 115–128. 12204303 10.1016/s0165-0270(02)00121-8

[39] Della-Maggiore V, Chau W, Peres-Neto PR, McIntosh AR (2002) An empirical comparison of SPM preprocessing parameters to the analysis of fMRI data. Neuroimage 17: 19–28. 12482065 10.1006/nimg.2002.1113

[40] Bennett CM, Wolford GL, Miller MB (2009) The principled control of false positives in neuroimaging. Soc Cogn Affect Neurosci 4: 417–422. 20042432 10.1093/scan/nsp053 PMC2799957

[41] Poldrack RA (2012) The future of fMRI in cognitive neuroscience. Neuroimage 62: 1216–1220. 21856431 10.1016/j.neuroimage.2011.08.007 PMC4131441

[42] Genovese CR, Lazar NA, Nichols T (2002) Thresholding of statistical maps in functional neuroimaging using the false discovery rate. Neuroimage 15: 870–878. 11906227 10.1006/nimg.2001.1037

[43] Nichols TE, Holmes AP (2002) Nonparametric permutation tests for functional neuroimaging: A primer with examples. Hum Brain Mapp 15: 1–25. 11747097 10.1002/hbm.1058 PMC6871862

[44] Nichols T, Hayasaka S (2003) Controlling the familywise error rate in functional neuroimaging: A comparative review. Stat Methods Med Res 12: 419–446. 14599004 10.1191/0962280203sm341ra

[45] Friston KJ, Holmes A, Poline JB, Price CJ, Frith CD (1996) Detecting activations in PET and fMRI: Levels of inference and power. Neuroimage 4: 223–235. 9345513 10.1006/nimg.1996.0074

[46] Maxwell SE (2004) The persistence of underpowered studies in psychological research: Causes, consequences, and remedies. Psychol Methods 9: 147–163. 15137886 10.1037/1082-989X.9.2.147

[47] Mumford JA (2012) A power calculation guide for fMRI studies. Soc Cogn Affect Neurosci 7: 738–742. 22641837 10.1093/scan/nss059 PMC3427872

[48] Mumford JA, Nichols TE (2008) Power calculation for group fMRI studies accounting for arbitrary design and temporal autocorrelation. Neuroimage 39: 261–268. 17919925 10.1016/j.neuroimage.2007.07.061 PMC2423281

[49] Frackowiak RSJ, Ashburner JT, Penny WD, Zeki S (2004) Random effects analysis (chapter 12). In: Ashburner J, Friston K, Penny W, editors. Human Brain Function. London, UK: Academic Press.

[50] Moher D, Jones A, Lepage L, CONSORT Grp (2001) Use of the CONSORT statement and quality of reports of randomized trials - A comparative before-and-after evaluation. Jama-Journal of the American Medical Association 285: 1992–1995. 10.1001/jama.285.15.1992 11308436

[51] Plint AC, Moher D, Morrison A, Schulz K, Altman DG, et al (2006) Does the CONSORT checklist improve the quality of reports of randomised controlled trials? A systematic review. Med J Aust 185: 263–267. 16948622 10.5694/j.1326-5377.2006.tb00557.x

[52] Alvarez F, Meyer N, Gourraud PA, Paul C (2009) CONSORT adoption and quality of reporting of randomized controlled trials: A systematic analysis in two dermatology journals. Br J Dermatol 161: 1159–1165. 19681881 10.1111/j.1365-2133.2009.09382.x

[53] Young EA, Breslau N (2004) Cortisol and catecholamines in posttraumatic stress disorder: An epidemiologic community study. Arch Gen Psychiatry 61: 394–401. 15066898 10.1001/archpsyc.61.4.394

[54] Moher D, Schulz KF, Simera I, Altman DG (2010) Guidance for developers of health research reporting guidelines. PLoS Med 7: e1000217. 20169112 10.1371/journal.pmed.1000217 PMC2821895

[55] Carp J (2012) On the plurality of (methodological) worlds: Estimating the analytic flexibility of FMRI experiments. Front Neurosci 6: 149. 23087605 10.3389/fnins.2012.00149 PMC3468892

[56] Carp J (2013) Better living through transparency: Improving the reproducibility of fMRI results through comprehensive methods reporting. Cogn Affect Behav Neurosci 13: 660–666. 23839069 10.3758/s13415-013-0188-0

[57] Ince DC, Hatton L, Graham-Cumming J (2012) The case for open computer programs. Nature 482: 485–488. 22358837 10.1038/nature10836

