# Supplementary material for: The Reporting of Observational Clinical Functional Magnetic Resonance Imaging Studies: A Systematic Review
Source: PLoS One. 2014 Apr 22;9(4):e94412. doi: 10.1371/journal.pone.0094412 (PMC3995931; doi:10.1371/journal.pone.0094412)
Supplement: Figure S1 — Flow Diagram of Citation Selection Process. (DOC) [file pone.0094412.s001.doc]

**Citations Identified by Initial Search Strategy**

(n=1196)

**76 Duplicates Removed**

**Unique Citations Remained**

(n=1120)

**20 Not Randomly Selected**

**Citations Excluded, with Reasons** (n=1000)

not met inclusion/exclusion criteria

without clinical population

only in abstract form

editorials, letters, comments and reviews

no full-text publication obtained

not observational

**Citations Randomly Selected to Assess Eligibility**

(n=1100)

**Random Sample for Final Review**

(n= 100)

Neuron (n=2)

Nature Neuroscience (n=1)

PNAS (n=4)

Brain (n=22)

Journal of Neuroscience (n=13)

Neuroimage (n=58)

**Figure1**. **Flow Diagram of Citation Selection Process**
