# Supplementary material for: The Reporting of Observational Clinical Functional Magnetic Resonance Imaging Studies: A Systematic Review
Source: PLoS One. 2014 Apr 22;9(4):e94412. doi: 10.1371/journal.pone.0094412 (PMC3995931; doi:10.1371/journal.pone.0094412)
Supplement: File S1 — Sample size calculation for estimating a single proportion with a level of confidence. (DOC) [file pone.0094412.s003.doc]

File S1: Sample Size Calculation for Estimating a Single Proportion with a Level of Confidence

The primary outcomes of this paper are the proportion of reviewed articles that reported each item of the STROBE checklist and the proportion that reported estimates of parameters needed for future sample size determination. Given no prior similar studies have provided the estimates of the proportion, we determine sample sizes by varying the proportion estimates and margin of error over its plausible ranges (See the results below) through sensitivity analysis. The mathematics formula for sample size calculation with an expected estimate and precision is as follows:

The estimated range of $p$is $p$±$Z_{1-\frac{\alpha}{2}}\sqrt{\frac{p\times(1-p)}{n}}$, MOE=$Z_{1-\frac{\alpha}{2}}\sqrt{\frac{p\times(1-p)}{n}}$, then $n=\frac{Z_{1-\frac{\alpha}{2}}^{2}\times p(1-p)}{MOE^{2}}$

As shown in the table below, we notice that the sample size of 96 can achieve any estimate of proportion of reporting at a MOE of 10% and also can reach its extreme estimates of proportion with a value less than 5% or greater than 95% and with an MOE of 5% at a 95% confidence level. We therefore chose a sample size of 100 by rounding up from 96.

Sample Size Calculations by Varying Estimated Proportion and Margin of Error

| Estimated % of reporting ($p$) | Margin of Error (MOE) | |
| --- | --- | --- |
|  | 5% | 10% |
| 5% | 73 | 18 |
| 10% | 138 | 35 |
| 15% | 196 | 49 |
| 20% | 246 | 61 |
| 25% | 288 | 72 |
| 30% | 323 | 81 |
| 35% | 350 | 87 |
| 40% | 369 | 92 |
| 45% | 380 | 95 |
| 50% | 384 | 96 |
| 55% | 380 | 95 |
| 60% | 369 | 92 |
| 65% | 350 | 87 |
| 70% | 323 | 81 |
| 75% | 288 | 72 |
| 80% | 246 | 61 |
| 85% | 196 | 49 |
| 90% | 138 | 35 |
| 95% | 73 | 18 |
