# Supplementary material for: The Reporting of Observational Clinical Functional Magnetic Resonance Imaging Studies: A Systematic Review
Source: PLoS One. 2014 Apr 22;9(4):e94412. doi: 10.1371/journal.pone.0094412 (PMC3995931; doi:10.1371/journal.pone.0094412)
Supplement: File S2 — Sample size calculation for estimating a Cohen's kappa coefficient with a given precision. (DOC) [file pone.0094412.s004.doc]

File S2: Sample Size Calculation for Estimating a Kappa ($\kappa$) **with a Given Precision**

Assuming that the expected inter-reviewer agreement (Kappa’s coefficient) is$\kappa$, the expected proportion of agreements by chance is$p_{e}$and the observed proportion of agreements is$p_{0}$. If there are $n$observations, then a 95% confidence interval for $\kappa$is calculated as follows [30]:

$\kappa-Z_{1-\frac{\alpha}{2}}se(\kappa)$ $to$ $\kappa+Z_{1-\frac{\alpha}{2}}se(\kappa)$, $where$ $se(\kappa)=\sqrt{\frac{p_{0}(1-p_{0})}{n(1-p_{e})^{2}}}$, $\kappa=\frac{p_{0}-p_{e}}{1-p_{e}}$

$$MOE=Z_{1-\frac{\alpha}{2}}se(\kappa)=Z_{1-\frac{\alpha}{2}}\sqrt{\frac{p_{0}(1-p_{0})}{n(1-p_{e})^{2}}}$$

With estimates of$\kappa$, $MOE$and$p_{e}$, the mathematical formula for sample size determination is given by:

$$n=\frac{Z_{1-\frac{\alpha}{2}}^{2}[\kappa+(1-\kappa)p_{e}](1-\kappa)}{MOE^{2}(1-p_{e})}$$

Over the plausible ranges of$\kappa$, $p_{e}$ and$MOE$, the sample sizes are determined through sensitivity analysis with results shown in the table below. Under the assumptions that the MOE is 30%, we calculate a sample size around 49 to achieve an estimate of Kappa’s coefficient greater than or equal to 0.4 at$p_{e}$≤ 0.6, with a 95% confidence interval. Therefore we chose a sample size of 50 by rounding up from 49.

Sample Size Calculations by Varying Estimated Kappa’s Coefficient and Expected Proportion of Agreements by Chance at the Margin of Error of 30%

| $p_{e}$ | Kappa’s Coefficient $\kappa$ | | | | | | | |
| --- | --- | --- | --- | --- | --- | --- | --- | --- |
|  | 0.4 | 0.5 | 0.6 | 0.7 | | 0.8 | | 0.9 |
|  |  |  | MOE=30% | |  | |  |  |
|  | n | *n* | *n* | *n* | | *n* | | *n* |
| 0.05 | 12 | 12 | 11 | 10 | | 7 | | 4 |
| 0.1 | 13 | 13 | 12 | 10 | | 8 | | 4 |
| 0.2 | 17 | 16 | 15 | 12 | | 9 | | 5 |
| 0.3 | 21 | 20 | 18 | 14 | | 10 | | 6 |
| 0.4 | 27 | 25 | 22 | 17 | | 13 | | 7 |
| 0.5 | 36 | 32 | 27 | 22 | | 15 | | 8 |
| 0.6 | 49 | 43 | 36 | 28 | | 20 | | 10 |
| 0.7 | 70 | 60 | 50 | 39 | | 27 | | 14 |
| 0.8 | 113 | 96 | 79 | 60 | | 41 | | 21 |
| 0.9 | 241 | 203 | 164 | 124 | | 84 | | 42 |
