# Supplementary material for: The Reporting of Observational Clinical Functional Magnetic Resonance Imaging Studies: A Systematic Review
Source: PLoS One. 2014 Apr 22;9(4):e94412. doi: 10.1371/journal.pone.0094412 (PMC3995931; doi:10.1371/journal.pone.0094412)
Supplement: File S3 — List of 100 eligible studies. (DOC) [file pone.0094412.s005.doc]

File S3: Reference of 100 Eligible Articles

1. Alvarez F, Meyer N, Gourraud PA, Paul C (2009) CONSORT adoption and quality of reporting of randomized controlled trials: A systematic analysis in two dermatology journals. Br J Dermatol 161: 1159-1165.

2. Begg C, Cho M, Eastwood S, Horton R, Moher D, et al. (1996) Improving the quality of reporting of randomized controlled trials. the CONSORT statement. JAMA 276: 637-639.

3. Bennett CM, Wolford GL, Miller MB (2009) The principled control of false positives in neuroimaging. Soc Cogn Affect Neurosci 4: 417-422.

4. Birken CS, Parkin PC (1999) In which journals will pediatricians find the best evidence for clinical practice? Pediatrics 103: 941-947.

5. Bruer JT (1982) Methodological rigor and citation frequency in patient compliance literature. Am J Public Health 72: 1119-1123.

6. Carp J (2013) Better living through transparency: Improving the reproducibility of fMRI results through comprehensive methods reporting. Cogn Affect Behav Neurosci 13: 660-666.

7. Carp J (2012) The secret lives of experiments: Methods reporting in the fMRI literature. Neuroimage 63: 289-300.

8. Carp J (2012) On the plurality of (methodological) worlds: Estimating the analytic flexibility of FMRI experiments. Front Neurosci 6: 149.

9. Carter CS, Heckers S, Nichols T, Pine DS, Strother S (2008) Optimizing the design and analysis of clinical functional magnetic resonance imaging research studies. Biol Psychiatry 64: 842-849.

10. Chan AW, Altman DG (2005) Epidemiology and reporting of randomised trials published in PubMed journals. Lancet 365: 1159-1162.

11. Chan AW, Altman DG (2005) Identifying outcome reporting bias in randomised trials on PubMed: Review of publications and survey of authors. BMJ 330: 753.

12. Chan AW, Hrobjartsson A, Jorgensen KJ, Gotzsche PC, Altman DG (2008) Discrepancies in sample size calculations and data analyses reported in randomised trials: Comparison of publications with protocols. BMJ 337: a2299.

13. Chan AW, Krleza-Jeric K, Schmid I, Altman DG (2004) Outcome reporting bias in randomized trials funded by the canadian institutes of health research. CMAJ 171: 735-740.

14. Charles P, Giraudeau B, Dechartres A, Baron G, Ravaud P (2009) Reporting of sample size calculation in randomised controlled trials: Review. BMJ 338: b1732.

15. Churchill NW, Oder A, Abdi H, Tam F, Lee W, et al. (2012) Optimizing preprocessing and analysis pipelines for single-subject fMRI. I. standard temporal motion and physiological noise correction methods. Hum Brain Mapp 33: 609-627.

16. Clopper C, Pearson ES (1934) The use of confidence or fiducial limits illustrated in the case of the binomial. Biometrika 26: 404-413.

17. Dale AM (1999) Optimal experimental design for event-related fMRI. Hum Brain Mapp 8: 109-114.

18. Della-Maggiore V, Chau W, Peres-Neto PR, McIntosh AR (2002) An empirical comparison of SPM preprocessing parameters to the analysis of fMRI data. Neuroimage 17: 19-28.

19. Dwan K, Altman DG, Arnaiz JA, Bloom J, Chan AW, et al. (2008) Systematic review of the empirical evidence of study publication bias and outcome reporting bias. PLoS One 3: e3081.

20. El Emam K, Jonker E, Arbuckle L, Malin B (2011) A systematic review of re-identification attacks on health data. 12 (6).

21. Frackowiak RSJ, Ashburner JT, Penny WD, Zeki S (2004) Random effects analysis (chapter 12). In: Ashburner J, Friston K, Penny W, editors. Human Brain Function. London, UK: Academic Press.

22. Friston KJ, Holmes A, Poline JB, Price CJ, Frith CD (1996) Detecting activations in PET and fMRI: Levels of inference and power. Neuroimage 4: 223-235.

23. Friston KJ, Holmes AP, Price CJ, Buchel C, Worsley KJ (1999) Multisubject fMRI studies and conjunction analyses. Neuroimage 10: 385-396.

24. Friston KJ, Holmes AP, Worsley KJ (1999) How many subjects constitute a study? Neuroimage 10: 1-5.

25. Genovese CR, Lazar NA, Nichols T (2002) Thresholding of statistical maps in functional neuroimaging using the false discovery rate. Neuroimage 15: 870-878.

26. Glahn DC, Ragland JD, Abramoff A, Barrett J, Laird AR, et al. (2005) Beyond hypofrontality: A quantitative meta-analysis of functional neuroimaging studies of working memory in schizophrenia. Hum Brain Mapp 25: 60-69.

27. Hopfinger JB, Buchel C, Holmes AP, Friston KJ (2000) A study of analysis parameters that influence the sensitivity of event-related fMRI analyses. Neuroimage 11: 326-333.

28. Huang W, Pach D, Napadow V, Park K, Long X, et al. (2012) Characterizing acupuncture stimuli using brain imaging with FMRI--a systematic review and meta-analysis of the literature. PLoS One 7: e32960.

29. Huettel SA, Song AW, and McCarthy G (2009) Functional magnetic resonance imaging : Sunderland, MA: Sinauer Associates, Inc.

30. Ince DC, Hatton L, Graham-Cumming J (2012) The case for open computer programs. Nature 482: 485-488.

31. Langan S, Schmitt J, Coenraads PJ, Svensson A, von Elm E, et al. (2010) The reporting of observational research studies in dermatology journals: A literature-based study. Arch Dermatol 146: 534-541.

32. Lee KP, Schotland M, Bacchetti P, Bero LA (2002) Association of journal quality indicators with methodological quality of clinical research articles. JAMA 287: 2805-2808.

33. MacDonald AW,3rd, Thermenos HW, Barch DM, Seidman LJ (2009) Imaging genetic liability to schizophrenia: Systematic review of FMRI studies of patients' nonpsychotic relatives. Schizophr Bull 35: 1142-1162.

34. Maxwell SE (2004) The persistence of underpowered studies in psychological research: Causes, consequences, and remedies. Psychol Methods 9: 147-163.

35. Moher D, Jones A, Lepage L, CONSORT Grp (2001) Use of the CONSORT statement and quality of reports of randomized trials - A comparative before-and-after evaluation. Jama-Journal of the American Medical Association 285: 1992-1995.

36. Moher D, Schulz KF, Simera I, Altman DG (2010) Guidance for developers of health research reporting guidelines. PLoS Med 7: e1000217.

37. Monk CS, Klein RG, Telzer EH, Schroth EA, Mannuzza S, et al. (2008) Amygdala and nucleus accumbens activation to emotional facial expressions in children and adolescents at risk for major depression. Am J Psychiatry 165: 90-98.

38. Mumford JA (2012) A power calculation guide for fMRI studies. Soc Cogn Affect Neurosci 7: 738-742.

39. Nichols T, Hayasaka S (2003) Controlling the familywise error rate in functional neuroimaging: A comparative review. Stat Methods Med Res 12: 419-446.

40. Nichols TE, Holmes AP (2002) Nonparametric permutation tests for functional neuroimaging: A primer with examples. Hum Brain Mapp 15: 1-25.

41. Opthof T (1997) Sense and nonsense about the impact factor. Cardiovasc Res 33: 1-7.

42. Papathanasiou AA, Zintzaras E (2010) Assessing the quality of reporting of observational studies in cancer. Ann Epidemiol 20: 67-73.

43. Plint AC, Moher D, Morrison A, Schulz K, Altman DG, et al. (2006) Does the CONSORT checklist improve the quality of reports of randomised controlled trials? A systematic review. Med J Aust 185: 263-267.

44. Poldrack RA (2012) The future of fMRI in cognitive neuroscience. Neuroimage 62: 1216-1220.

45. Poldrack RA, Fletcher PC, Henson RN, Worsley KJ, Brett M, et al. (2008) Guidelines for reporting an fMRI study. Neuroimage 40: 409-414.

46. Schoonbaert D, Roelants G (1996) Citation analysis for measuring the value of scientific publications: Quality assessment tool or comedy of errors? Trop Med Int Health 1: 739-752.

47. Sheline YI, Barch DM, Donnelly JM, Ollinger JM, Snyder AZ, et al. (2001) Increased amygdala response to masked emotional faces in depressed subjects resolves with antidepressant treatment: An fMRI study. Biol Psychiatry 50: 651-658.

48. Shoukri MM (2011) Measures of interobserver agreement. : Boca Raton, Fla:Chapman & Hall/CRC.

49. Siegle GJ, Steinhauer SR, Thase ME, Stenger VA, Carter CS (2002) Can't shake that feeling: Event-related fMRI assessment of sustained amygdala activity in response to emotional information in depressed individuals. Biol Psychiatry 51: 693-707.

50. Simera I, Moher D, Hoey J, Schulz KF, Altman DG (2010) A catalogue of reporting guidelines for health research. Eur J Clin Invest 40: 35-53.

51. Skudlarski P, Constable RT, Gore JC (1999) ROC analysis of statistical methods used in functional MRI: Individual subjects. Neuroimage 9: 311-329.

52. Snitz BE, MacDonald A,3rd, Cohen JD, Cho RY, Becker T, et al. (2005) Lateral and medial hypofrontality in first-episode schizophrenia: Functional activity in a medication-naive state and effects of short-term atypical antipsychotic treatment. Am J Psychiatry 162: 2322-2329.

53. Strother S, La Conte S, Kai Hansen L, Anderson J, Zhang J, et al. (2004) Optimizing the fMRI data-processing pipeline using prediction and reproducibility performance metrics: I. A preliminary group analysis. Neuroimage 23, Supplement 1: S196-S207.

54. von Elm E, Altman DG, Egger M, Pocock SJ, Gotzsche PC, et al. (2007) The strengthening the reporting of observational studies in epidemiology (STROBE) statement: Guidelines for reporting observational studies. Ann Intern Med 147: 573-577.

55. Worsley KJ (2005) Spatial smoothing of autocorrelations to control the degrees of freedom in fMRI analysis. Neuroimage 26: 635-641.

56. Worsley KJ (2003) Detecting activation in fMRI data. Stat Methods Med Res 12: 401-418.

57. Worsley KJ (1997) An overview and some new developments in the statistical analysis of PET and fMRI data. Hum Brain Mapp 5: 254-258.

58. Worsley KJ, Cao J, Paus T, Petrides M, Evans AC (1998) Applications of random field theory to functional connectivity. Hum Brain Mapp 6: 364-367.

59. Worsley KJ, Liao CH, Aston J, Petre V, Duncan GH, et al. (2002) A general statistical analysis for fMRI data. Neuroimage 15: 1-15.

60. Yoon JH, Minzenberg MJ, Ursu S, Ryan Walter BS, Wendelken C, et al. (2008) Association of dorsolateral prefrontal cortex dysfunction with disrupted coordinated brain activity in schizophrenia: Relationship with impaired cognition, behavioral disorganization, and global function. Am J Psychiatry 165: 1006-1014.

61. Young EA, Breslau N (2004) Cortisol and catecholamines in posttraumatic stress disorder: An epidemiologic community study. Arch Gen Psychiatry 61: 394-401.

62. Young NS, Ioannidis JP, Al-Ubaydli O (2008) Why current publication practices may distort science. PLoS Med 5: e201.
