# Supplementary material for: The Reporting of Observational Clinical Functional Magnetic Resonance Imaging Studies: A Systematic Review
Source: PLoS One. 2014 Apr 22;9(4):e94412. doi: 10.1371/journal.pone.0094412 (PMC3995931; doi:10.1371/journal.pone.0094412)
Supplement: Table S1 — Search strategy for Ovid Medline database. (DOC) [file pone.0094412.s007.doc]

Table S1

Database: Ovid MEDLINE(R) in-Process & Other Non-indexed Citations and Ovid MEDLINE (R) 1948 – Present. This search was conducted on February 12, 2012*

| Step Number | Search Strategy |
| --- | --- |
| 1 | functional magnetic resonance imaging.mp. |
| 2 | fmri.mp. |
| 3 | 1 or 2 |
| 4 | limit 3 to (english language and humans and yr="2010-2011") |
| 5 | "neuron".jn. |
| 6 | "nature neuroscience".jn. |
| 7 | “proceedings of the national academy of science of the united states of america”.jn. |
| 8 | ”brain”.jn. |
| 9 | ”journal of neuroscience”.jn. |
| 10 | "neuroimage".jn. |
| 11 | 4 and 5 |
| 12 | 4 and 6 |
| 13 | 4 and 7 |
| 14 | 4 and 8 |
| 15 | 4 and 9 |
| 16 | 4 and 10 |
| 17 | 11 or 12 or 13 or 14 or 15 or 16 |

** This search was limited to the years 2010-2011, six journals, English language and humans
