# Supplementary material for: The Reporting of Observational Clinical Functional Magnetic Resonance Imaging Studies: A Systematic Review
Source: PLoS One. 2014 Apr 22;9(4):e94412. doi: 10.1371/journal.pone.0094412 (PMC3995931; doi:10.1371/journal.pone.0094412)
Supplement: Table S2 — Data extraction form containing 83 items adapted from Poldrack et al.'s checklist. (DOC) [file pone.0094412.s008.doc]

**Table S2:** Data extraction form containing 83 items adapted from Poldrack et al.’s checklist. Three items dropped from the checklist are indicated. Information extracted from each eligible article. The coding for the article is entered in the column, namely “Article#”†.

| Category | Item No | Item Description | Instructions | Article# |
| --- | --- | --- | --- | --- |
| EXPERIMENTAL DESIGN -design specification | 1a | Describe number of blocks, trials, experimental units per session or per subject |  |  |
|  | 1b | State length of each trial and interval between trials |  |  |
|  | 1c | If ISIs are variable, report the mean and range of ISIs and how they are distributed | If ISIs are constant, it should be recorded ‘Not Applicable’. |  |
|  | 1d | *Block-Designs*: specify the length of blocks | If not a block design, it should be recorded ‘Not Applicable’. |  |
|  | 1e | *Event-related Designs*: state whether the design is optimized for efficiency, and if so, state how | If not an event-related design, it should be recorded ‘Not Applicable’. |  |
|  | 1f | *Mixed designs*: state correlation between block and event regressors | If not a mixed design, it should be recorded ‘Not Applicable’. |  |
|  | 1g | Instructions: state what subjects are asked to do |  |  |
| EXPERIMENTAL DESIGN - task specification | 2a | Stimuli: state whether specific stimuli repeated across trials |  |  |
|  | 2b | Stimuli: describe what the Stimuli are and how many there are |  |  |
|  | 2c | Stimuli: state whether specific stimuli repeated across trials |  |  |
| EXPERIMENTAL DESIGN - planned comparison | 3 | If the experiment has multiple conditions, state what the specific planned comparisons are, or whether an omnibus ANOVA test is used |  |  |
| HUMAN SUBJECTS - details on subject sample | 4a | State number of subjects |  |  |
|  | 4b | State age (mean and range) |  |  |
|  | 4c | State handedness |  |  |
|  | 4d | State number of males or females |  |  |
|  | 4e | State inclusion and exclusion criteria, |  |  |
|  | 4f | If any subjects were scanned but then rejected from analysis after data collection, state how many and reasons for rejection | Report in either methods or results section |  |
|  | 4g | For group comparisons, state what variables (if any) were equated across groups. |  |  |
| HUMAN SUBJECTS - ethics approval | 5 | State which Institutional Review Board (IRB) approved the protocol |  |  |
| HUMAN SUBJECTS - behavioral performance | 6 | State how behavioral performance was measured (e.g., response time, accuracy) |  |  |
| DATA ACQUISITION - image properties | 7a | Describe manufacturer, field strength (in Tesla), model name |  |  |
|  | 7b | State the number of experimental sessions and volumes acquired per session |  |  |
|  | 7c | State pulse sequence type (gradient/spin echo, EPI/spiral) |  |  |
|  | 7d | State field of view, matrix size, slice thickness, inter-slice skip |  |  |
|  | 7e | State acquisition orientation (axial, sagittal, coronal, oblique; if axials co-planar with AC-PC, the volume coverage in terms of Z in mm) |  |  |
|  | 7f | State clearly whether it is on the whole brain. If not, state area of acquisition |  |  |
|  | 7g | State order of acquisition of slices (sequential or interleaved) |  |  |
|  | 7h | State TE, TR, flip angle |  |  |
| DATA ACQUISITION - data preprocessing | 8a | For each piece of software used, give the version number. If no version number is available, date of last application of updates) |  |  |
|  | 8b | If any subjects required different processing operations or settings in the analysis, those differences should be specified explicitly |  |  |
| DATA ACQUISITION - preprocessing general | 9a | Specify order of preprocessing operations |  |  |
|  |  | Describe any data quality control measures | *Deleted |  |
|  |  | Unwarping of B0 distortions | *Deleted |  |
|  | 9b | Slice timing correction: reference slice and type of interpolation used (e.g., “Slice timing correction to the first slice as performed, using SPM5’s Fourier phase shift interpolation”) |  |  |
|  | 9c | Motion correction: reference scan, image similarity metric, type of interpolation used, degrees-of-freedom (If not rigid body) and, ideally, optimization method |  |  |
| DATA ACQUISITION - intersubject registration | 10a | Illustrate the voxels present in all subjects using mask image |  |  |
|  | 10b | Describe transformation model (linear/affine, nonlinear), type of any non-linear transformations (polynomial, discrete cosine basis), number of parameters (e.g., 12 parameter affine), regularization image-similarity metric, and interpolation method |  |  |
|  | 10c | State object image information (image used to determine transformation to atlas) |  |  |
|  | 10d | State if anatomical MRI is co-planar with functional acquisition |  |  |
|  | 10e | State whether functional acquisition is co-registered to anatomical |  |  |
|  | 10f | If functional acquisition is co-registered to anatomical, state how (such as segmented gray image or functional image) |  |  |
|  | 10g | State Atlas/target information |  |  |
|  | 10h | State brain image template space, name, modality and resolution (e.g, “FSL’s MNI Avg 152, T1 2x2x2 mm”; “SPM2’s MNI gray matter template 2x2x2 mm”) |  |  |
|  | 10i | State typically MNI, Talairach, or MNI converted to Talairach. |  |  |
|  | 10j | If MNI is converted to Talairach, state the method used (e.g., Brett’s mni2tal) |  |  |
|  | 10k | State clearly how anatomical locations (e.g., gyral anatomy, Brodmann areas) were determined (e.g., paper atlas, Talairach Daemon, manual inspection of individuals’ anatomy, etc.) |  |  |
| DATA ACQUISITION - smoothing | 11 | Describe size and type of smoothing kernel (e.g., for a group study, “12 mm FHWM Gaussian smoothing applied to ameliorate differences in inter-subject localization”; for single subject fMRI “6 mm FWHM Gaussian smoothing used to reduce noise”) |  |  |
| STATISTICAL MODELING - general issues | 12 | For novel methods that are not described in detail in a separate paper, provide explicit description and validation of method either in the text or as an appendix |  |  |
| STATISTICAL MODELING - intrasubject fMRI modeling info | 13a | Describe statistical model and estimation method: multiple regression is most common statistical model; estimation methods are typically ordinary least squares (OLS), OLS with adjustment for autocorrelation |  |  |
|  | 13b | State block/epoch-based or event-related model |  |  |
|  | 13c | Specify hemodynamic response function (HRF): assumed HRF model, HRF basis, or estimated HRF |  |  |
|  | 13d | Clearly state additional regressors used (e.g., temporal derivatives, motion, behavioral covariates) |  |  |
|  | 13e | State any orthogonalization of regressors |  |  |
|  | 13f | State the drift modeling or high-pass filtering (e.g., “DCT with cut off of X seconds”; “Gaussian-weighted running line smoother, cut-off 100 seconds”, or “cubic polynomial”) |  |  |
|  | 13g | Describe the autocorrelation model type, and whether global or local |  |  |
|  | 13h | State contrast construction: exactly what terms are subtracted from? Define these in terms of task or stimulus conditions |  |  |
| STATISTICAL MODELING - group modeling info | 14a | State statistical model, estimation method, and inference type |  |  |
|  | 14b | If fixed effects inference used, provide the justification |  |  |
|  | 14c | If more than 2-levels, describe the levels and assumptions of the model (eg, are variances assumed equal between groups) |  |  |
|  | 14d | State if there are repeated measures. If multiple measurements per subject, list method to account for within subject correlation, exact assumptions made about correlation and variance. |  |  |
| STATISTICAL INFERENCE - inference on statistic image (thresholding) | 15a | State type of search region for analysis, and the volume in voxels or CC |  |  |
|  | 15b | If not whole brain, state how region was determined; method for constructing region should be independent of present statistic image |  |  |
|  | 15c | If threshold used for inference and threshold used for visualization in figures is different, clearly state so and list each | If thresholds for inference and visualization were the same, we should record ‘Not Applicable’. |  |
|  | 15d | Explicitly state if inferences are corrected for multiple comparisons |  |  |
|  | 15e | If correction is limited to a small volume, the method for selecting the region should be stated explicitly |  |  |
|  | 15f | If no formal multiple comparisons method is used, the inference must be explicitly labeled “uncorrected” |  |  |
|  | 15g | Describe if it is voxel-wise significance |  |  |
|  | 15h | State if inferences are corrected for Family-wise error (FWE) or false discovery rate (FDR) |  |  |
|  | 15i | If FWE found by random field theory list the smoothness in mm FWHM and the RESEL count |  |  |
|  | 15j | If FWE found by simulation (eg, AFNI AlphaSim), provide details of parameters for simulation |  |  |
|  | 15k | If not a standard method, specify the method for finding significance |  |  |
|  | 15l | State cluster-defining threshold (eg, *P*=*0.001*) |  |  |
|  | 15m | State the corrected cluster significance level (e.g., “Statistic images were assessed for cluster-wise significance using a cluster-defining threshold of *P*=0.001; the 0.05 FWE-corrected critical cluster size was 103”) |  |  |
|  | 15n | If significance determined with random field theory, then smoothness and RESEL count must be supplied |  |  |
|  | 15o | State correction for multiple planned comparisons based upon each voxel |  |  |
|  | 15p | State observed effect size for any failure to reject the null hypothesis (e.g., lack of activation in a particular region) |  |  |
| STATISTICAL INFERENCE - ROI analysis | 16a | Describe how ROIs were defined (eg, functional versus anatomical localizer) |  |  |
|  | 16b | Describe how signal was extracted within ROI (e.g., average parameter estimates, FIR deconvolution) |  |  |
|  | 16c | If percent signal change reported, describe how scaling factor was determined (eg, height of block regressor or height of isolated event regressor) |  |  |
|  | 16d | State if percent signal change is relative to voxel-mean, or whole-brain mean |  |  |
| FIGURES AND TABLES - general | 17a | State the statistical map that the figure or table is based upon (e.g., *Z*, *t*, *p*) |  |  |
|  | 17b | Provide the thresholds used to created the image or figure (intensity and cluster extent, where appropriate) |  |  |
| FIGURES AND TABLES - figures | 18 | State the underlying anatomical image (e.g., average anatomy, template image) |  |  |
|  |  | State any additional operations (e.g., masking out parts of the image) | *Deleted |  |
| FIGURES AND TABLES - tables | 19a | Provide locations in stereotactic space (with the space described specifically) |  |  |
|  | 19b | Provide statistics for each cluster (including maximum and cluster extent) |  |  |
|  | 19c | Provide source of anatomical labels (eg, atlas, automated labeling method) |  |  |

† Coding for each evaluated item: 0 – “Not Reported”, 1 – “Reported”, 2 – “Not Applicable”

* Deleted from the Poldrack et al.’s checklist due to substantial subjectivity

Abbreviations: ISIs, inter-stimulus intervals; ANOVA, analysis of variance; EPI, [Echo Planar Imaging](http://www.mr-tip.com/serv1.php?type=db1&dbs=Echo Planar Imaging); TE, echo time; TR, repetition time; MRI, magnetic resonance imaging; MNI, Montreal Neurological Institute space; DCT, discrete cosine transform; CC, cubic centimeter; FWE, family-wise error; FDR, false discovery rate; FWHM, full-width at half-maximum; RESEL, resolution element; ROI, region of interest; FIR, finite impulse response
